# Geometric property of off resonance error robust composite pulse

**DOI:** 10.1038/s41598-022-13207-z

**Published:** 2022-06-10

**Authors:** Shingo Kukita, Haruki Kiya, Yasushi Kondo

**Affiliations:** Department of Physics, Kindai University, Higashi, Osaka 577-8502 Japan

**Keywords:** Quantum information, Quantum mechanics, Qubits, Theoretical physics

## Abstract

The precision of quantum operations is affected by unavoidable systematic errors. A composite pulse (CP), which has been well investigated in nuclear magnetic resonance (NMR), is a technique that suppresses the influence of systematic errors by replacing a single operation with a sequence of operations. In one-qubit operations, there are two typical systematic errors, Pulse Length Error (PLE) and Off Resonance Error (ORE). Recently, it was found that PLE robust CPs have a clear geometric property. In this study, we show that ORE robust CPs also have a simple geometric property, which is associated with trajectories on the Bloch sphere of the corresponding operations. We discuss the geometric property of ORE robust CPs using two examples.

## Introduction

Quantum technologies that utilise quantum states, such as quantum computing^1–3^ and metrology^4–6^, require precise control of the states. However, the quantum systems that we are attempting to control basically experience unavoidable environmental noise and systematic errors caused by experimental apparatuses. They prevent us from precise control of quantum states.

To suppress the effects of systematic errors, a composite pulse (CP) has been investigated^7–9^ particularly in the field of nuclear magnetic resonance (NMR), which can be utilised to implement toy quantum computers^10–12^. In CPs, a single operation is replaced by a sequence of operations, and the error in each operation cancels each other up to a certain order with respect to the error magnitude. In this paper, we focus on first-order CPs, which compensate for first-order effects of error magnitude. The effectiveness of CPs has been exhibited in NMR^13^, ion traps^14^, and superconducting circuits^15^.

In NMR, two types of errors, Pulse Length Error (PLE) and Off Resonance Error (ORE), in one-qubit operations have been intensively studied. If we adopt the Bloch sphere representation, in which a state of a qubit is represented by a point in a three-dimensional sphere (Bloch sphere) while an operation by a rotation, PLE corresponds to the error of the rotational angle of an operation, and ORE corresponds to the error of the rotation axis. Many CPs that are robust against PLE, such as BB1^16^, SCROFULOUS^17^ and SK1^18^, have been found. Similarly, we have many construction methods for CPs that are robust against ORE^8,19–23^. When we perform a specific target operation, such as $\pi$- and $\pi /2$-rotations in the Bloch sphere representation, these methods work well and can provide simple and explicit formulae for determining ORE robust operations. The CORPSE family is one of the simplest and most tractable ORE robust CPs for implementing an arbitrary $\theta$-rotation^24^; all of its parameters are explicitly determined as a simple function of the parameters of the target operation. This family is often used when ORE robust arbitrary $\theta$-rotations are required^25–28^.

Recently, it was revealed that PLE robust CPs have a geometric property related to the concept of the geometric quantum gates^29^. Additionally, a geometric meaning of robustness against any type of noise is discussed in Ref.^30^; however, it is associated with the geometry of Lie algebras and abstract. A concrete geometric property of ORE robust CPs has not yet been addressed.

In this study, we investigated a geometric property of ORE robust CPs via geometry on the Bloch sphere. We obtained the relation between the ORE robustness of a CP and its trajectory. This geometric property will provide a deeper understanding of the ORE robustness and increase its tractability. Further, we illustrated this property using two ORE robust CPs, CORPSE and another CP, which we have newly invented. For some simple cases, this property will help us to obtain new ORE robust CPs intuitively.

The remainder of this paper is organised as follows. We briefly review basic concepts of CPs in “Review of composite pulses”. In “Geometric property of ORE robust CP”, we explain our result on the geometric property of ORE robust gates. “Two examples of ORE robust composite pulses” introduces two ORE robust CPs, CORPSE and our proposed CP, and we examine how the geometric picture of ORE robust CPs works. “Conclusions and discussions” presents the conclusions and discussions. We use the natural unit $\hbar =1$ throughout the paper.

## Review of composite pulses

### General framework

Here, we introduce a general theory of CPs, which is applicable to any quantum systems although we will focus on two-level systems from the next subsection. The Schrödinger equation with a time-dependent Hamiltonian *H*(*t*) is

1 $$\begin{aligned} \frac{d}{d t}|\Psi (t)\rangle = - i H(t) |\Psi (t)\rangle . \end{aligned}$$

The formal solution from $t=0$ to $t=T$ in the Schrödinger picture is given by

2 $$\begin{aligned} |\Psi (T)\rangle = \mathcal{T}\exp \Bigl (- i \int ^{T}_{0} d t H(t)\Bigr ) |\Psi (0)\rangle , \end{aligned}$$

where we introduce the symbol of the time-ordered product:

3 $$\begin{aligned} \mathcal{T}(A(t_{1})B(t_{2}))= {\left\{ \begin{array}{ll} A(t_{1})B(t_{2})\quad t_{1}>t_{2},\\ B(t_{2})A(t_{1})\quad t_{2}>t_{1}. \end{array}\right. } \end{aligned}$$

Its extension to cases of multiple operators is trivial. If the Hamiltonian is piecewise constant, the time-ordered exponential can also be rewritten as

4 $$\begin{aligned} \mathcal{T}\exp \Bigl (-i \int ^{T}_{0} d t H(t)\Bigr )=\exp \bigl (-i (t_{k}-t_{k-1}) H_{k}\bigr )\exp \bigl (-i (t_{k-1}-t_{k-2}) H_{k-1}\bigr ){\cdot\cdot\cdot} \exp \bigl (-i (t_{1}-t_{0}) H_{1}\bigr ), \end{aligned}$$

where we set $t_{0}=0$ and $t_{k}=T$ and the Hamiltonian is

5 $$\begin{aligned} H(t)=H_{i},~t_{i-1} \le t < t_{i},~(i=1 \sim k). \end{aligned}$$

*k* is the number of periods in which the Hamiltonian is constant. We refer to an operation during the Hamiltonian is constant as an elementary operation. Then, *k* represents the number of elementary operations.

Let us assume that the Hamiltonian is decomposed into two parts:

6 $$\begin{aligned} H(t)=H_{0}(t)+ H_{\mathrm{err}}(t). \end{aligned}$$

The part $H_{0}(t)$ represents the ideal Hamiltonian, whose corresponding dynamics $U_{0}(T,0):=\mathcal{T}\exp (-i \int ^{T}_{0}d t H_{0}(t))$ will be implemented herein. We can control this part of the Hamiltonian. Meanwhile, $H_{\mathrm{err}}(t)$ describes the effect of undesired systematic errors during the operation. The magnitude of $H_{\mathrm{err}}(t)$ is assumed to be sufficiently small compared to that of $H_{0}(t)$ for the entire time region. (Mathematically, all the matrix components of $H_{\mathrm{err}}(t)$ are small compared to that of $H_{0}(t)$.) Either part $H_{0}(t)$ or $H_{\mathrm{err}}(t)$ can have (piecewise) time dependence. We define the state in the interaction picture with respect to $H_{0}(t)$ as

7 $$\begin{aligned} |\Psi _{I}(t)\rangle := \Bigl ( \mathcal{T}\exp \Bigl (- i \int ^{t}_{0} d t' H_{0}(t')\Bigr ) \Bigr )^{\dagger }|\Psi (t) \rangle =U^{\dagger }_{0}(t,0)|\Psi (t)\rangle , \end{aligned}$$

where $|\Psi (t)\rangle$ is a solution to the Schrödinger equation (1). The state $|\Psi _{I}(t)\rangle$ satisfies the following equation:

8 $$\begin{aligned} \frac{d}{d t}|\Psi _{I}(t)\rangle =- i {\tilde{H}}_{\mathrm{err}}(t)|\Psi _{I}(t)\rangle , \end{aligned}$$

where ${\tilde{H}}_{\mathrm{err}}(t):=U^{\dagger }_{0}(t,0)H_{\mathrm{err}}(t)U_{0}(t,0)$. A formal solution of Eq. (8) is

9 $$\begin{aligned} |\Psi _{I}(T)\rangle ={\tilde{U}}_{\mathrm{err}}(T,0)|\Psi _{I}(0)\rangle := \mathcal{T}\exp \Bigl (- i \int ^{T}_{0} d t {\tilde{H}}_{\mathrm{err}}(t)\Bigr ) |\Psi _{I}(0)\rangle . \end{aligned}$$

By comparing the above Eq. (9) and the definition of the interaction picture (7), the solution of the Schrödinger equation with the Hamiltonian (6) is written as

10 $$\begin{aligned} |\Psi (T)\rangle =U_{0}(T,0)|\Psi _{I}(T)\rangle =U_{0}(T,0){\tilde{U}}_{\mathrm{err}}(T,0)|\Psi (0)\rangle . \end{aligned}$$

We intend to implement the ideal unitary operation $U_{0}(T,0)$ as accurately as possible under the effect of the error term $H_{\mathrm{err}}$. As the magnitude of $H_{\mathrm{err}}$ is assumed to be small enough, we can ignore its higher-order terms in ${\tilde{U}}_{\mathrm{err}}(T,0)$, and then

11 $$\begin{aligned} {\tilde{U}}_{\mathrm{err}}(T,0) \sim \Bigl (1- i \int ^{T}_{0} d t {\tilde{H}}_{\mathrm{err}}(t)\Bigr ). \end{aligned}$$

When the condition

12 $$\begin{aligned} \int ^{T}_{0} d t {\tilde{H}}_{\mathrm{err}}(t)=0. \end{aligned}$$

is satisfied, the operation (10) equals the ideal operation $U_{0}(T,0)$ for any initial state $|\Psi (0)\rangle$ up to the first order with respect to ${\tilde{H}}_{\mathrm{err}}(t)$. If better accuracy is required, we obtain more conditions from higher-order terms. Note that to implement a unitary evolution *U* as the target operation, there are many choices of $H_{0}(t)$ that satisfy $U=U_{0}(T,0)$. If we know the error model $H_{\mathrm{err}}(t)$, we can take $H_{0}(t)$ to satisfy $U=U_{0}(T,0)$ and Eq. (12) using the degrees of freedom of this choice of $H_{0}(t)$. Thus, the operation caused by such a Hamiltonian $H_{0}(t)$ traces the unitary operation *U* in a robust manner against the specific error model $H_{\mathrm{err}}(t)$. This is called a CP, one of several methods compensating for the effect of specific errors, such as adiabatic pulses, shortcuts to adiabaticity, and optimal control.

### Qubit control

The most fundamental quantum system in quantum information processing is a two-level system, called a qubit. Hereinafter, we focus on the control of one qubit. The control Hamiltonian of a qubit is given as

13 $$\begin{aligned} H_{0}(t)= \omega (t)\vec {n}(t)\cdot \frac{\vec {\sigma }}{2}:= \omega (t)(n_{x}(t)\frac{\sigma _{x}}{2}+n_{y}(t)\frac{\sigma _{y}}{2}+n_{z}(t)\frac{\sigma _{z}}{2}), \end{aligned}$$

where the vector $\vec {n}(t)=(n_{x},n_{y},n_{z})$ is a time-dependent unit vector, and $\vec {\sigma }:=(\sigma _{x},\sigma _{y},\sigma _{z})$ are the Pauli matrices:

14 $$\begin{aligned} \sigma _{x}= \begin{pmatrix} 0&{}1\\ 1&{}0 \end{pmatrix} ,~ \sigma _{y}= \begin{pmatrix} 0&{}-i\\ i&{}0 \end{pmatrix} ,~ \sigma _{z}= \begin{pmatrix} 1&{}0\\ 0&{}-1 \end{pmatrix} . \end{aligned}$$

We implement an arbitrary control of a qubit by adjusting $\omega (t)$ and $\vec {n}(t)$. The time dependence of these controllable parameters is often piecewise constant, as shown in Eq. (5), which corresponds to a sequence of pulses. Note that any state of a qubit can be mapped to a point on a sphere (called the Bloch sphere). In this representation, we map the eigenvectors of $\sigma _{z}$, $|\uparrow \rangle :=(1,0)^{t}$ and $|\downarrow \rangle :=(0,1)^{t}$ to the north pole $\vec {z}=(0,0,1)^{t}$ and the south pole $-\vec {z}=(0,0,-1)^{t}$ on the Bloch sphere, respectively. The superscript *t* means the matrix transposition. Accordingly, the operation of the qubit by the Hamiltonian (13) with constant $\omega$ and $\vec {n}$ corresponds to the rotation with the rotation angle $\theta :=\omega \tau$ and the axis $\vec {n}$, where $\tau$ is a period during which $\omega$ and $\vec {n}$ are applied. Hereinafter, we consider the case where the Hamiltonian is piecewise constant.

When we consider the control of one qubit, there are two types of systematic errors: PLE and ORE.

#### PLE case

PLE is described by the change in $\omega (t)$ in the control Hamiltonian, $\omega (t) \rightarrow \omega (t)(1+\epsilon )$, where $\epsilon$ is a small parameter representing the magnitude of PLE. We should comment that the name of PLE might not be appropriate. This error has two origins: imperfection of the pulse (control field) amplitude and pulse duration. Because pulse duration has less imperfection in NMR, the error in the amplitude is the dominant origin of PLE. As the name of pulse *length* error rather sounds to be caused by the pulse duration error, we may be ought to call it pulse amplitude error. However, in this paper, we call this error PLE following the fashion in NMR.

The control Hamiltonian $H_{0}(t)$ and the error Hamiltonian $H_{\mathrm{err}}(t)$ are given by

15 $$\begin{aligned} H_{0}(t)=\omega _{i}\vec {n}_{i}\cdot \vec {\sigma }/2,\quad H_{\mathrm{err}}(t)=\epsilon \omega _{i}\vec {n}_{i}\cdot \vec {\sigma }/2,\quad t_{i-1}<t<t_{i}. \end{aligned}$$

Here, we assume that $\epsilon$ is constant at $0<t<T$ because it represents a systematic error. In this case, $U_{0}(t,0)$ and ${\tilde{H}}_{\mathrm{err}}$ are given as

16 $$\begin{aligned} U_{0}(t,0)=&e^{-i\omega _{i}(t-t_{i-1})\vec {n}_{i}\cdot \vec {\sigma }/2} V_{i-1} {\cdot\cdot\cdot} V_{0},\quad t_{i-1}<t<t_{i},\nonumber \\ {\tilde{H}}_{\mathrm{err}}(t)=&U^{\dagger }_{0}(t,0)(\epsilon \omega _{i}\vec {n}_{i}\cdot \vec {\sigma }/2)U_{0}(t,0)=\epsilon \omega _{i} V^{\dagger }_{0}{\cdot\cdot\cdot} V^{\dagger }_{i-1} (\vec {n}_{i}\cdot \vec {\sigma }/2)V_{i-1}{\cdot\cdot\cdot} V_{0},\quad t_{i-1}<t<t_{i}, \end{aligned}$$

where $V_{i}$ denotes $\exp (-i \theta _{i}\vec {n}_{i}\cdot \vec {\sigma }/2)$ while $V_{0}$ is the identity matrix and $\theta _{i}:=\omega _{i}(t_{i}-t_{i-1})$. $V_{0}$ is inserted in the last line just for ensuring the consistency with the range of $i=1\sim k$. The condition of error robustness (12) for PLE is

17 $$\begin{aligned} \int ^{T}_{0} d t {\tilde{H}}_{\mathrm{err}}(t)=&\bigg ( \int ^{t_{1}}_{0}d t+\int ^{t_{2}}_{t_{1}}d t {\cdot\cdot\cdot} +\int ^{T}_{t_{k-1}}d t\biggr ){\tilde{H}}_{\mathrm{err}}(t)\nonumber \\ =&\epsilon \int ^{t_{1}}_{0} d t (\omega _{1}\vec {n}_{1}\cdot \vec {\sigma })+\epsilon \int ^{t_{2}}_{t_{1}} d t V^{\dagger }_{1}(\omega _{2}\vec {n}_{2}\cdot \vec {\sigma })V_{1}+{\cdot\cdot\cdot} +\epsilon \int ^{T}_{t_{k-1}} d t V^{\dagger }_{1}{\cdot\cdot\cdot} V_{k-1}(\omega _{k}\vec {n}_{k}\cdot \vec {\sigma }/2)V_{k-1}{\cdot\cdot\cdot} V_{1}\nonumber \\ =&\epsilon \omega _{1}t_{1}(\vec {n}_{1}\cdot \vec {\sigma }/2)+ \epsilon \omega _{2}(t_{2}-t_{1})V^{\dagger }_{1} (\vec {n}_{2}\cdot \vec {\sigma }/2) V_{1}+{\cdot\cdot\cdot} + \epsilon \omega _{k} (T-t_{k-1}) V^{\dagger }_{1}{\cdot\cdot\cdot} V^{\dagger }_{k-1} (\vec {n}_{k}\cdot \vec {\sigma }/2)V_{k-1}{\cdot\cdot\cdot} V_{1} \nonumber \\ =&\epsilon \sum ^{k}_{i=1}V^{\dagger }_{0}{\cdot\cdot\cdot} V^{\dagger }_{i-1} (\theta _{i}\vec {n}_{i}\cdot \vec {\sigma }/2) V_{i-1}{\cdot\cdot\cdot} {\cdot\cdot\cdot} V_{0}=0. \nonumber \\ \end{aligned}$$

In Ref.^29^, it was shown that Eq. (17) means a vanishing total dynamic phase, and then it leads to a geometric quantum gate^31–33^ based on the concept of holonomy^34–37^. Many PLE robust CPs, such as BB1^16^, SCROFULOUS^17^, and SK1^18^, have been found to date.

#### ORE case

ORE is typically caused by the miscalibration of the resonance frequency of a qubit. When the quantisation axis is the *z*-axis of the Bloch sphere, the (piecewise constant) control Hamiltonian $H_{0}(t)$ and the error Hamiltonian $H_{\mathrm{err}}(t)$ are given by

18 $$\begin{aligned} H_{0}(t)=\omega _{i}\vec {n}_{i}\cdot \vec {\sigma }/2,\quad H_{\mathrm{err}}(t)=f \frac{\sigma _{z}}{2},\quad t_{i-1}<t<t_{i}. \end{aligned}$$

where *f* is a small parameter corresponding to ORE magnitude. Note that the error term for ORE is time independent. The error Hamiltonian in the interaction picture is

19 $$\begin{aligned} {\tilde{H}}_{\mathrm{err}}(t)= V^{\dagger }_{0}{\cdot\cdot\cdot} V^{\dagger }_{i-1}e^{i \omega _{i}(t-t_{i-1})\vec {n}_{i}\cdot \vec {\sigma }/2}\bigg (f\frac{\sigma _{z}}{2}\bigg )e^{-i\omega _{i} (t-t_{i-1})\vec {n}_{i}\cdot \vec {\sigma }/2}V_{i-1}{\cdot\cdot\cdot} V_{0},\quad t_{i-1}<t<t_{i}, \end{aligned}$$

where we use the same definition of $V_{i}$ as Eq. (16). Note that $e^{-i \omega _{i}(t-t_{i-1})\vec {n}_{i}\cdot \vec {\sigma }/2}$ does not commute with $H_{\mathrm{err}}(t)$ ($\sigma _{z}$ in this case) unlike the case of PLE. Then the condition (12) is calculated as

20 $$\begin{aligned} \int ^{T}_{0} d t {\tilde{H}}_{\mathrm{err}}(t)&=f\int ^{t_{1}}_{0} d t e^{i \omega _{1}t \vec {n}_{1}\cdot \vec {\sigma }/2 } \frac{\sigma _{z}}{2} e^{-i \omega _{1}t \vec {n}_{1}\cdot \vec {\sigma }/2 } \nonumber \\&\quad + f\int ^{t_{2}}_{t_{1}} d t V^{\dagger }_{1}e^{i \omega _{2}(t-t_{1}) \vec {n}_{2}\cdot \vec {\sigma }/2 } \frac{\sigma _{z}}{2} e^{-i \omega _{2} (t-t_{1}) \vec {n}_{2}\cdot \vec {\sigma }/2}V_{1}+{\cdot\cdot\cdot} \nonumber \\&\quad +f\int ^{T}_{t_{k-1}} d t V^{\dagger }_{1}{\cdot\cdot\cdot} V^{\dagger }_{k-1} e^{i \omega _{k}(t-t_{k-1}) \vec {n}_{k}\cdot \vec {\sigma }/2} \frac{\sigma _{z}}{2} e^{-i \omega _{k}(t-t_{k-1}) \vec {n}_{k}\cdot \vec {\sigma }/2}V_{k-1}{\cdot\cdot\cdot} V_{1}\nonumber \\&=f\sum ^{k}_{i=1}V^{\dagger }_{0}{\cdot\cdot\cdot} V^{\dagger }_{i-1} \Bigl (\int ^{t_{i}}_{t_{i-1}} d t e^{i \omega _{i} (t-t_{i-1}) \vec {n}_{1}\cdot \vec {\sigma }/2 }\frac{\sigma _{z}}{2}e^{-i \omega _{i}(t-t_{i-1}) \vec {n}_{1}\cdot \vec {\sigma }/2 }\Bigr ) V_{i-1}{\cdot\cdot\cdot} V_{0}=0. \end{aligned}$$

This ORE robustness condition seems to be more complicated than that of PLE (17). However, in this study, we show that this condition has a simple geometric understanding via geometry on the Bloch sphere.

CORPSE, which was proposed in Ref.^24^, is a well known ORE robust CP. This CP can perform an arbitrary $\theta$ rotation in an ORE robust manner with considerably simple operations. Each parameter in the CORPSE sequence is explicitly determined as a function of the parameters of the target operation.

## Geometric property of ORE robust CP

### General case

Here, we explain a geometric property of Eq. (20). First, note that for any 3-dimensional unit vector $\vec {n}$ and $\vec {m}$,

21 $$\begin{aligned} e^{-i \theta \vec {m}\cdot \vec {\sigma }/2} (\vec {n}\cdot \vec {\sigma }) e^{i \theta \vec {m}\cdot \vec {\sigma }/2}=\bigl ({{\varvec{R}}}(\theta ,\vec {m})\vec {n} \bigr )\cdot \vec {\sigma }, \end{aligned}$$

where ${{\varvec{R}}}(\theta ,\vec {m})$ is a $3 \times 3$ rotation matrix with rotational axis $\vec {m}$ and angle $\theta$. Using this equality, the right hand side of Eq. (20) can be rewritten as

22 $$\begin{aligned} & f\sum ^{k}_{i=1}V^{\dagger }_{0}{\cdot\cdot\cdot} V^{\dagger }_{i-1} \Bigl (\int ^{t_{i}}_{t_{i-1}} d t e^{i \omega _{i}(t-t_{i-1}) \vec {n}_{1}\cdot \vec {\sigma }/2 }\frac{\sigma _{z}}{2}e^{-i \omega _{i}(t-t_{i-1}) \vec {n}_{1}\cdot \vec {\sigma }/2 }\Bigr ) V_{i-1}{\cdot\cdot\cdot} V_{0}\nonumber \\&\quad =f\sum ^{k}_{i=1}V^{\dagger }_{0}{\cdot\cdot\cdot} V^{\dagger }_{i-1} \Bigl (\int ^{t_{i}}_{t_{i-1}} d t e^{i \omega _{i}(t-t_{i-1}) \vec {n}_{1}\cdot \vec {\sigma }/2 } (\vec {z} \cdot \frac{\vec {\sigma }}{2}) e^{-i \omega _{i}(t-t_{i-1}) \vec {n}_{1}\cdot \vec {\sigma }/2 }\Bigr ) V_{i-1}{\cdot\cdot\cdot} V_{0}\nonumber \\&\quad =f\Biggl (\sum ^{k}_{i=1} {{\varvec{R}}}^{-1}(\theta _{1},\vec {n}_{1}) {{\varvec{R}}}^{-1}(\theta _{2},\vec {n}_{2}) {\cdot\cdot\cdot} {{\varvec{R}}}^{-1}(\theta _{i-1},\vec {n}_{i-1}) \int ^{t_{i}}_{t_{i-1}} d t {{\varvec{R}}}^{-1}( \omega _{i}(t-t_{i-1}),\vec {n}_{i})\vec {z}\Biggr )\cdot \frac{\vec {\sigma }}{2}. \end{aligned}$$

Thus, we obtain the following condition from Eq. (20),

23 $$\begin{aligned} \sum ^{k}_{i=1} \Bigl ({{\varvec{R}}}^{-1}(\theta _{1},\vec {n}_{1}) {{\varvec{R}}}^{-1}(\theta _{2},\vec {n}_{2}) {\cdot\cdot\cdot} {{\varvec{R}}}^{-1}(\theta _{i-1},\vec {n}_{i-1}) \int ^{t_{i}}_{t_{i-1}} d t {{\varvec{R}}}^{-1}( \omega _{i}(t-t_{i-1}),\vec {n}_{i})\vec {z}\Bigr )=\vec {0}. \end{aligned}$$

This condition is also equivalent to the following for any vector $\vec {p}\in {{\mathbb {R}}}^{3}$:

24 $$\begin{aligned}& \vec {p}^{~t}\cdot \Biggl (\sum ^{k}_{i=1} \Bigl ({{\boldsymbol{R}}}^{-1}(\theta _{1},\vec {n}_{1}) {{\boldsymbol{R}}}^{-1}(\theta _{2},\vec {n}_{2}) {\cdot\cdot\cdot} {{\boldsymbol{R}}}^{-1}(\theta _{i-1},\vec {n}_{i-1}) \int ^{t_{i}}_{t_{i-1}} d t {{\boldsymbol{R}}}^{-1}( \omega _{i}(t-t_{i-1}),\vec {n}_{i})\vec {z}\Bigr )\Biggr )=0, \\&\quad \Longleftrightarrow \vec {z}^{~t}\cdot \Biggl (\sum ^{k}_{i=1} \int ^{t_{i}}_{t_{i-1}} d t {{\boldsymbol{R}}}( \omega _{i}(t-t_{i-1}),\vec {n}_{i})\vec {p}_{i-1}\Biggr )=0, \end{aligned}$$

where $\vec {p}_{i}:={{\varvec{R}}}(\theta _{i},\vec {n}_{i}){\cdot\cdot\cdot} {{\varvec{R}}}(\theta _{1},\vec {n}_{1}) \vec {p}$ with $\vec {p}_{0}:=\vec {p}$. In the second line, we transpose the equation and use ${{\varvec{R}}}^{t}(\theta ,\vec {m})={{\varvec{R}}}^{-1}(\theta ,\vec {m})$. As the above equation is linear, we can normalise the length of $\vec {p}$ without loss of generality, and then we identify $\vec {p}$ with a position vector (quantum state) on the Bloch sphere. Let ${{\mathbb {R}}}^{3}_{n}$ denote the space of the position vectors on the Bloch sphere.

$\vec {p}_{i}$ represents the point after the sequence of rotations ${{\varvec{R}}}(\theta _{i},\vec {n}_{i}){{\varvec{R}}}(\theta _{i-1},\vec {n}_{i-1}){\cdot\cdot\cdot} {{\varvec{R}}}(\theta _{1},\vec {n}_{1})$. By introducing $\vec {p}(t):= {{\varvec{R}}}(\omega _{i}(t-t_{i-1}),\vec {n}_{i}) p_{i-1}~(t_{i-1}\le t < t_{i})$, we further summarise Eq. (24) as

25 $$\begin{aligned} \vec {z}^{~t}\cdot \int ^{T}_{0} d t \vec {p}(t)=0,~~\forall \vec {p}\in {{\mathbb {R}}}^{3}_{n}. \end{aligned}$$

The integration in the above equation is the sum of all position vectors on the trajectory,

26 $$\begin{aligned} \vec {p}(t):~\vec {p}\xrightarrow {{{\varvec{R}}}(\theta _{1},\vec {n}_{1})}\vec {p}_{1} \xrightarrow {{{\varvec{R}}}(\theta _{2},\vec {n}_{2})} {\cdot\cdot\cdot} \xrightarrow {{{\varvec{R}}}(\theta _{k},\vec {n}_{k})} \vec {p}_{k}. \end{aligned}$$

**Figure 1:**
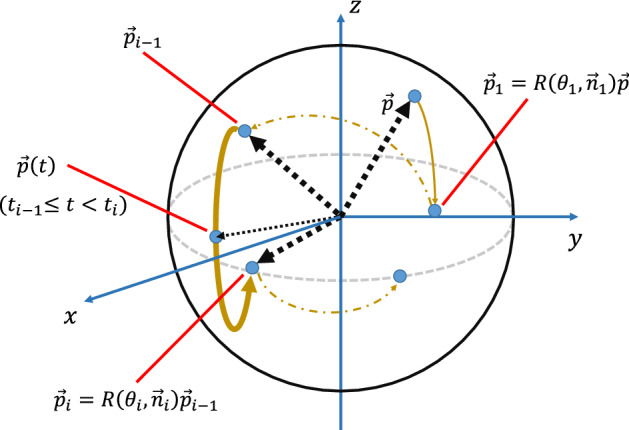
Schematic picture of the trajectory of $\vec {p}$ by ${{\varvec{R}}}(\theta _{k},\vec {n}_{k}){\cdot\cdot\cdot} {{\varvec{R}}}(\theta _{1},\vec {n}_{1})$ on the Bloch sphere. The thick curve represents the trajectory of the *i*-th operation from $\vec {p}_{i-1}$ to $\vec {p}_{i}$. The *i*-th part of the summation in Eq. (24) is the integration of all position vectors along this trajectory.

See Fig. 1. This sequence of rotations corresponds to the action of the errorless unitary operation $V_{k}{\cdot\cdot\cdot} V_{1}$ in the Bloch sphere representation. Accordingly, $\vec {p}(t)~(0<t<T)$ represents the trajectory of the initial state $\vec {p}$ by the action of this unitary operation. This leads to the following geometrical expression of the ORE robustness condition: *a CP is ORE robust if and only if, for any initial state *$\vec {p}$,*the time integral of all position vectors on the trajectory by this CP in the Bloch sphere representation exists on the xy plane.* Thus, we now express the ORE robustness condition of a CP via the geometric concept associated with the trajectory by the CP. Note that this geometric property is also valid when the Hamiltonian is not piecewise constant but continuously time-dependent; intuitively, the continuous case corresponds to the limit, $k\rightarrow \infty$ and $t_{i}-t_{i-1}\rightarrow 0$, while keeping $T=\sum ^{k}_{i=1} (t_{i}-t_{i-1})$ constant.

### Constant-$\omega$ case

Here, we consider the case where $\omega _{i}$, the magnitude of the control field $H_{0}$, is constant throughout the operation: $\omega _{i}=\omega ~(0<t<T)$, which is a common assumption in quantum information processing. In the Bloch sphere representation, a constant $\omega$ means a constant angular velocity of the motion of the position vector representing a quantum state. We can set $\omega =1$ without loss of generality. Under this assumption, the above definition (25) of the ORE robustness condition can further be interpreted in a purely geometric way.

First, we focus on each part of the sum in Eq. (24):

27 $$\begin{aligned} \int ^{t_{i}}_{t_{i-1}} d t {{\varvec{R}}}( t-t_{i-1},\vec {n}_{i})\vec {p}_{i-1}, \end{aligned}$$

which represents the integral of all the position vectors on the trajectory from the time $t_{i-1}$ to $t_{i}$. The position vector *p*(*t*) during this time interval sweeps the trajectory from $p_{i-1}$ to $p_{i}$ with a constant speed because both the angular velocity and the rotation radius are constant. The constant rotation radius follows from the constant Hamiltonian during $t_{i-1}$ and $t_{i}$: The dynamics in the Bloch sphere by the Hamiltonian during this period is a simple rotation with a constant radius. Note that the sweeping speed can differ for different time intervals, e.g., $t_{1}\rightarrow t_{2}$ and $t_{2}\rightarrow t_{3}$ because the rotation radii are not necessarily the same.

Equation (27) is rewritten as

28 $$\begin{aligned} \int ^{t_{i}}_{t_{i-1}} d t {{\varvec{R}}}( t-t_{i-1},\vec {n}_{i})\vec {p}_{i-1}=(t_{i}-t_{i-1})\frac{\int ^{t_{i}}_{t_{i-1}} d t {{\varvec{R}}}( t-t_{i-1},\vec {n}_{i})\vec {p}_{i-1}}{t_{i}-t_{i-1}}=(t_{i}-t_{i-1})\vec {M}^{i}_{\vec {p}}=\theta _{i}\vec {M}^{i}_{\vec {p}}. \end{aligned}$$

Here we use $\omega =1$ and $\omega (t_{i}-t_{i-1})=\theta _{i}$, and $\vec {M}^{i}_{\vec {p}}$ can be identified as the geometric centre of all the position vectors $\vec {p}(t)$ during the interval from $t_{i-1}$ to $t_{i}$ because of the constant speed of $\vec {p}(t)$. More physically, this geometric centre corresponds to the mass centre of this arc on the trajectory when we assign a constant mass density to each point on the arc. We define $\theta _{i}$ as the mass of this arc. Thus, Eq. (28) can be regarded as (mass of this arc) $\times$ (mass centre of this arc).

The sum in Eq. (24) is correspondingly rewritten as

29 $$\begin{aligned} \sum ^{k}_{i=1} \int ^{t_{i}}_{t_{i-1}} d t {{\varvec{R}}}( t-t_{i-1},\vec {n}_{i})\vec {p}_{i-1}=\sum ^{k}_{i=1}\theta _{i}\vec {M}^{i}_{\vec {p}}. \end{aligned}$$

To further consider this sum of the vector, we recall the mass centre for a composite system that consists of *k* subsystems. When *i*-th subsystem has the mass $m_{i}$ and the mass centre $\vec {M}_{i}$, the mass centre $\vec {M}$ of the entire composite system is calculated as $\vec {M}=\sum ^{k}_{i=1}m_{i}\vec {M}_{i}/m$, where *m* is the total mass $\sum ^{k}_{i=1}m_{i}$. Thus, we define the mass centre of all the position vectors on the trajectory as

30 $$\begin{aligned} \vec {M}_{\vec {p}}:=\frac{\sum ^{k}_{i=1}\theta _{i}\vec {M}^{i}_{\vec {p}}}{\sum ^{k}_{i=1}\theta _{i}}. \end{aligned}$$

Note that the mass $\sum ^{k}_{i=1}\theta _{i}$ equals to the operation time *T*. Substituting this definition and Eq. (29) into Eq. (24), we obtain the following simple equation:

31 $$\begin{aligned} \vec {z}^{~t}\cdot \vec {M}_{\vec {p}}=0,~~~\forall \vec {p}\in {{\mathbb {R}}}^{3}_{n}. \end{aligned}$$

where we use the fact that $\sum ^{k}_{i=1}\theta _{i}=T$ is non-zero. This immediately implies that *a CP is ORE robust if and only if the mass centre of the errorless trajectory by this CP on the Bloch sphere exists on the xy plane for any initial state *$\vec {p}$. This is the geometric property of ORE robust CPs when we assume that $\omega$ is constant. In this case, all the information for calculating the ORE robustness is extracted purely graphically from the trajectories on the Bloch sphere.

## Two examples of ORE robust composite pulses

In this section, we verify that the mass centre of the trajectory lies on the *xy* plane for two ORE robust CPs: CORPSE and our newly proposed CP. First, we explain the notation. We assume that the unit vector $\vec {n}$ for each operation $e^{-i \theta \vec {n}\cdot \vec {\sigma }/2}$ is directed into the *xy* plane. Without loss of generality, we can represent $\vec {n}$ by $\vec {n}_{\phi }:=(\cos \phi ,\sin \phi ,0)$. We parametrise an operation by $\theta$ and $\phi$, and thus define $(\theta )_{\phi }:=e^{-i \theta \vec {n}_{\phi }\cdot \vec {\sigma }/2}$. Accordingly, a CP is written as a sequence $(\theta _{k})_{\phi _{k}}(\theta _{k-1})_{\phi _{k-1}}{\cdot\cdot\cdot} (\theta _{1})_{\phi _{1}}$. Also, we assume that $\omega =1$ during the entire operation. Hence, the discussion below is understood using the mass centre of trajectories, which is a purely geometric concept.

### CORPSE

A well-known ORE robust CP, CORPSE, can implement $(\theta )_{\phi }$ as the target operation for any $\theta$ and $\phi$. The CORPSE sequence of implementing $(\theta )_{\phi }$ comprises three elementary operations $(k=3)$ given by the following parameters:

32 $$\begin{aligned} \theta _{1}= 2 n_{1} \pi +\theta /2-\kappa ,~\theta _{2}= 2 n_{2} \pi -2 \kappa ,~\theta _{3}= 2 n_{3} \pi +\theta /2-\kappa ,~~\phi _{1}=\phi _{2}-\pi =\phi _{3}=\phi , \end{aligned}$$

where $\kappa =\arcsin (\sin (\theta /2)/2)$ and $n_{i}$ ($i=1,2,3$) are integers that satisfy $n_{1},n_{3} \ge 0$, and $n_{2}\ge 1$. Simple algebraic calculations show that the CORPSE sequence $(\theta _{3})_{\phi _{3}}(\theta _{2})_{\phi _{2}}(\theta _{1})_{\phi _{1}}$ satisfies $(\theta )_{\phi }=(\theta _{3})_{\phi _{3}}(\theta _{2})_{\phi _{2}}(\theta _{1})_{\phi _{1}}$ and is ORE robust.

**Figure 2:**
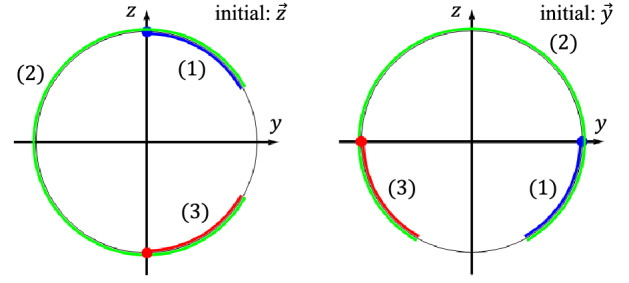
Errorless trajectories on the *yz* plane by CORPSE $(\frac{\pi }{3})_{0}(\frac{5\pi }{3})_{\pi }(\frac{\pi }{3})_{0}$, which performs $(\pi )_{0}$. The left (right) panel has $\vec {z}$ ($\vec {y}$) as the initial state. The initial (final) states are represented by the blue (red) points. The blue, green, and red curves represent the first, second, and third pulse trajectories, respectively. The left (right) panel has $\vec {z}$ ($\vec {y}$) as the initial state.

We show the ORE robustness of CORPSE from a geometric viewpoint. For simplicity, we consider the case in which CORPSE implement $(\pi )_{0}$, that is, the $\pi$ rotation with the direction $\vec {x}:=(1,0,0)$. Also, we take $n_{1}=n_{3}=0$ and $n_{2}=1$. The parameters for this case are $\theta _{1}=\theta _{3}=\pi /3$, $\theta _{2}=5 \pi /3$, $\phi _{1}=\phi _{3}=0$, and $\phi _{2}=\pi$. First, we note that it is only necessary to consider three linear independent unit vectors as $\vec {p}$ to examine the condition (31) owing to the linearity. We take $\vec {x}=(1,0,0)^{t}$, $\vec {y}=(0,1,0)^{t}$, $\vec {z}=(0,0,1)^{t}$ as $\vec {p}$. The vector $\vec {x}$ is a fixed point during the entire operation, and thus, the mass centre of the trajectory starting from $\vec {x}$ is on the *xy* plane. We then examine the trajectory from the staring points $\vec {y}$ and $\vec {z}$. It is convenient to show the *yz* plane to consider the mass centre of these trajectory (Fig. 2). Note that the information of the *x* direction is redundant when we consider these trajectories by CORPSE. The mass centre of the trajectory from $\vec {z}$ is calculated as follows.

33 $$\begin{aligned} \vec {M}_{\vec {z}}=&\frac{1}{T}\int ^{T}_{0} d t \vec {p}(t)=\frac{3}{7\pi }\int ^{\frac{7\pi }{3}}_{0} d t \vec {p}(t)\nonumber \\ =&\frac{3}{7\pi }\int ^{\frac{\pi }{3}}_{0} d \theta {{\varvec{R}}}(\theta ,\vec {x})(0,0,1)^{t}+ \frac{3}{7\pi }\int ^{\frac{5\pi }{3}}_{0} d \theta {{\varvec{R}}}(\theta ,-\vec {x})\Bigl (0,\sin \bigl (\frac{\pi }{3}\bigr ),\cos \bigl (\frac{\pi }{3}\bigr )\Bigr )^{t} +\frac{3}{7\pi }\int ^{\frac{\pi }{3}}_{0} d \theta {{\varvec{R}}}(\theta ,\vec {x})\Bigl (0,\sin \bigl (\frac{2\pi }{3}\bigr ),\cos \bigl (\frac{2\pi }{3}\bigr )\Bigr )^{t}\nonumber \\ =&\frac{3}{7\pi }\Biggl (\int ^{\frac{\pi }{3}}_{0}-\int ^{\frac{2\pi }{3}}_{\frac{\pi }{3}}+\int ^{\pi }_{\frac{2\pi }{3}}\Biggr ) d \theta (0,\sin \theta , \cos \theta )^{t}\nonumber \\ =&\frac{3}{7\pi }\Bigl ([(0,-\cos \theta , \sin \theta )^{t}]^{\frac{\pi }{3}}_{0}-[(0,-\cos \theta , \sin \theta )^{t}]^{\frac{2\pi }{3}}_{\frac{\pi }{3}}+[(0,-\cos \theta , \sin \theta )^{t}]^{\pi }_{\frac{2\pi }{3}}\Bigr )=\vec {0}. \end{aligned}$$

Thus $\vec {z}^{~t}\cdot \vec {M}_{\vec {z}}=\vec {z}^{~t}\cdot \vec {0}=0$. It is also shown that $\vec {M}_{\vec {y}}=\vec {0}$ because the trajectories from $\vec {y}$ and $\vec {z}$ (and these mass centres) are related by $\frac{\pi }{2}$-rotation. Refer to Fig. 2. Thus, the condition (31) is satisfied for CORPSE.

### CORP$^{2}$SE (Compensation for Off Resonance with a Perpendicularly combined Pulse SEquence)

CORPSE is a $k=3$ symmetric CP satisfying $\phi _{1}+\pi =\phi _{2}$ ($\vec {n}_{1}\cdot \vec {n}_{2}=-1$). It is a natural question whether there exists a symmetric $k=3$ CP with the condition $\phi _{1}+\pi /2=\phi _{2}$ ($\vec {n}_{1}\cdot \vec {n}_{2}=0$). Under the assumption that $\vec {n}_{1}\cdot \vec {n}_{2}=0$, we find the following symmetric $k=3$ CP that implements $(\theta )_{\phi }$:

34 $$\begin{aligned} \theta _{1}&=\theta _{3}=\arcsin \Bigl (-\sqrt{\frac{1-\alpha ^{2}}{1+\alpha ^{2}}}\Bigr ),~~\theta _{2}=\arccos (\alpha ^{2})\nonumber \\&\quad \phi _{1}+{3\pi /4}=\phi _{3}+{3\pi /4}=\phi _{2}{+}\pi /4=\phi , \end{aligned}$$

where $\alpha =\cos (\theta /2)$. We named this sequence CORP$^{2}$SE (*Compensation for Off Resonance with a Perpendicularly constructed Pulse SEquence*). CORPSE has the same rotation axis for all elementary operations whereas CORP$^{2}$SE has one orthogonal rotation axis at the middle operation. In Materials, we evaluate the performance of CORP$^{2}$SE comparing with CORPSE.

**Figure 3:**
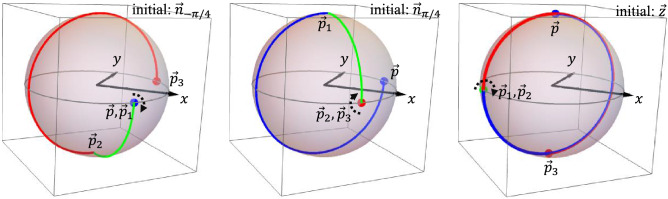
Errorless trajectories by CORP$^{2}$SE. The left, middle, and right panels show $\vec {n}_{-\pi /4}$, $\vec {n}_{\pi /4}$, and $\vec {z}$ as the initial state. The initial (final) states are represented by the blue (red) points. The blue, green, and red lines represent the first, second, and third pulse trajectories, respectively. The black arrows show the action of pulses when the state remains at a fixed point of the pulse.

We consider the case in which CORP$^{2}$SE (34) performs $(\pi )_{0}$. The parameters are taken to be $\theta _{1}=\theta _{3}=\frac{3 \pi }{2}$, $\theta _{2}=\pi /2$, $\phi _{1}=\phi _{3}=-\frac{3\pi }{4}$, and $\phi _{2}=-\frac{\pi }{4}$. In Fig. 3, we show the errorless trajectories from $\vec {z}$, $\vec {n}_{-\pi /4}=\frac{1}{\sqrt{2}}(1,-1,0)$, and $\vec {n}_{\pi /4}=\frac{1}{\sqrt{2}}(1,1,0)$, which are convenient to evaluate the mass centres of trajectories. Each trajectory has the same length on the northern and southern hemispheres. For instance, the trajectory from $\vec {z}$ (right panel) has a length of $3\times \pi /2$ for each hemisphere. As trajectories from all three initial states are always on great circles, or equivalently the rotation radius is always 1, the length is simply translated to the mass. That is, the northern and southern parts of the trajectory has the same mass. Also, one can graphically find that the mass centres of the northern and southern part have the same absolute value of the *z* component for all cases. These two facts implies that the mass centre of the trajectory is directed onto the *xy* plane for all initial states. Thus, for $\pi$-rotation, we graphically confirm that this CORP$^{2}$SE is ORE robust.

## Conclusions and discussions

In this study, we found the geometric property of the ORE robustness condition. We consider the position vector $\vec {p}(t)$ on the Bloch sphere, which represents a quantum state during a CP. The time average of $\vec {p}(t)$ for any initial state has a vanishing *z* component if and only if the CP is ORE robust. When the magnitude of the control Hamiltonian is constant, this integral of $\vec {p}(t)$ is proportional to the mass centre of the trajectory. For CORPSE and our proposed ORE robust CP, CORP$^{2}$SE, we confirmed that the mass centres of their trajectories from any initial point are on the *xy* plane on the Bloch sphere, as expected. Until now, only the geometric property of PLE robust pulses has been concretely known, and such properties of ORE robust CPs have not been well investigated. One reason for this is that the error term of ORE does not commute with the control Hamiltonian even at the same time unlike PLE. This makes the analysis of the ORE robust condition difficult algebraically. Our results of the geometric property will increase the tractability of ORE robust CPs and provide a deeper understanding of the ORE robustness. Also, the geometrical property of the ORE robustness could be utilized when we seek unknown ORE robust CPs. At least, we can intuitively (or without calculations) check whether an operation sequence is ORE robust or not, although direct calculations might be better when the sequence is complicated.

## Materials

### Performance comparison between CORPSE and CORP$^{2}$SE

**Figure 4:**
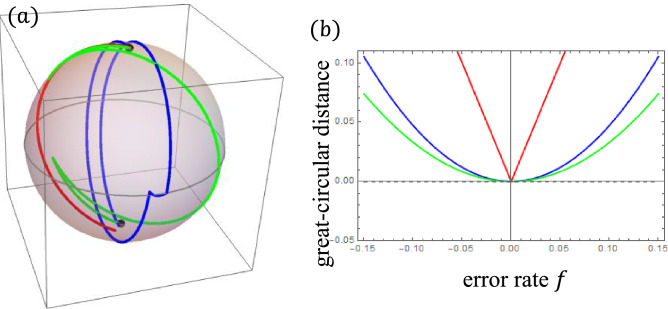
The performance of the ORE robust CPs. In (**a**), the trajectories with ORE for two CPs targeting the $(\pi )_{0}$ operation are drawn. The red, green, and blue lines represent the elementary $\pi$ operation, CORPSE, and CORP$^{2}$SE, respectively. The ORE rate is set to be $f=0.1$. The initial state is $\vec {z}$. In (**b**), we plot the accuracy of these operations defined by the great-circular distance as a function of the ORE rate *f*. The colours of the curves represent the same operations as those in (**a**).

We evaluate the performance of CORP$^{2}$SE and compare it with that of CORPSE. Figure 4 (a) shows the trajectories targeting the $(\pi )_{0}$ operation by them with ORE from the initial state $\vec {z}$. We can observe that both CPs compensate ORE, compared with the elementary $(\pi )_{0}$ operation.

To examine the performance of the CPs more precisely, we consider the great-circular distance on the Bloch sphere. For the $(\pi )_{0}$ operation on the state corresponding to $\vec {z}$ (north pole), the ideal operation maps the initial state $\vec {z}$ to $-\vec {z}$. The great-circular distance between $-\vec {z}$ and the end point of the operation with ORE from $\vec {z}$ has a positive value as a function of *f*. We characterise the accuracy of the $\pi$ rotation using the great-circular distance from $-\vec {z}$. The smaller the distance, the better the accuracy. Figure 4 (b) plots the great-circular distance between $-\vec {z}$ and the end point of the elementary $\pi$ operation, CORPSE, and CORP$^{2}$SE initiated from $\vec {z}$. While the absolute value of the error rate *f* increases, the distance increases for any case, and the accuracy of the $\pi$ operation decreases. The distance for CORPSE and CORP$^{2}$SE behaves as $\propto f^{2}$ around $f=0$, whereas the distance for the elementary $\pi$ operation behaves as $\propto |f|$. These behaviours are due to the definition of the ORE robustness. CORP$^{2}$SE provides slightly worse accuracy than CORPSE in the entire region of *f*, and the total CORP$^{2}$SE operation (non-dimensionalised) time is longer than that of CORPSE: $7 \pi /3$ for CORPSE and $7 \pi /2$ for CORP$^{2}$SE.
